# The coming era of a new auscultation system for analyzing respiratory sounds

**DOI:** 10.1186/s12890-022-01896-1

**Published:** 2022-03-31

**Authors:** Yoonjoo Kim, YunKyong Hyon, Sunju Lee, Seong-Dae Woo, Taeyoung Ha, Chaeuk Chung

**Affiliations:** 1 Division of Pulmonology and Critical Care Medicine, Department of Internal Medicine, College of Medicine, Chungnam National University, Daejeon, 34134 Korea; 2 Division of Industrial Mathematics, National Institute for Mathematical Sciences, 70, Yuseong-daero 1689 beon-gil, Yuseong-gu, Daejeon, 34047 Republic of Korea; 3 Infection Control Convergence Research Center, Chungnam National University School of Medicine, Daejeon, 35015 Republic of Korea

**Keywords:** Auscultation, Digital stethoscope, Deep learning, Artificial intelligence, Neural network, Wearable or wireless device

## Abstract

Auscultation with stethoscope has been an essential tool for diagnosing the patients with respiratory disease. Although auscultation is non-invasive, rapid, and inexpensive, it has intrinsic limitations such as inter-listener variability and subjectivity, and the examination must be performed face-to-face. Conventional stethoscope could not record the respiratory sounds, so it was impossible to share the sounds. Recent innovative digital stethoscopes have overcome the limitations and enabled clinicians to store and share the sounds for education and discussion. In particular, the recordable stethoscope made it possible to analyze breathing sounds using artificial intelligence, especially based on neural network. Deep learning-based analysis with an automatic feature extractor and convoluted neural network classifier has been applied for the accurate analysis of respiratory sounds. In addition, the current advances in battery technology, embedded processors with low power consumption, and integrated sensors make possible the development of wearable and wireless stethoscopes, which can help to examine patients living in areas of a shortage of doctors or those who need isolation. There are still challenges to overcome, such as the analysis of complex and mixed respiratory sounds and noise filtering, but continuous research and technological development will facilitate the transition to a new era of a wearable and smart stethoscope.

## Background

In the long-standing history of mankind, auscultation has long been widely used for the examination of patients [1]. A stethoscope is considered one of the most valuable medical devices because it is non-invasive, available in real-time, and much informative [2]. It is particularly useful in respiratory diseases, and abnormal respiratory sounds provide information on various pathological conditions of lungs and bronchi. In 1817, French doctor Rene Laennec invented an auscultation tool and it enabled him to listen to internal noises of patients [3, 4]. Since then, the stethoscope has gradually changed to a device with a binaural form, flexible tubing, and a rigid diaphragm [5, 6].

So far, the stethoscope has been widely used and adopted as the physician’s primary medical tool. However, as chest images are developed, the degree of dependence on auscultation is relatively decreasing [7]. This phenomenon may be caused by the inherent subjectivity. The ability to recognize and differentiate the abnormal sounds depends on the listener's experience and knowledge. This discrepancy can potentially lead to inaccurate diagnosis and mistreatment. To improve this problem, there have been efforts to implement a standardized system to record and share lung sounds to analyze them accurately. Recent technical advances have allowed the recording of lung sounds with a digital stethoscope by electronical intensification of the sounds, and the sharing of recorded sound via blue-tooth transmission [6]. Besides, there have been published studies on artificial intelligence (AI)-assisted auscultation which recognizes the pattern of sounds and identifies their abnormalities, and some digital stethoscopes already adopted machine learning (ML) algorithms [8–25].

Another drawback of auscultation is the impossibility of remote care. When doctors examine patients with a stethoscope, auscultation must be implemented by contacting the stethoscope on the body of patients. Many patients with chronic diseases or limited mobility stay in nursing facilities or at home often without a medical practitioner [24, 25]. Moreover, the demand of patients in hard-to-reach area for telemedicine is increasing nowadays. However, it is difficult for doctors to examine these patients and auscultation is hardly done. Advances in battery technology developed embedded processors with low power consumption and integrated sensors to make stethoscopes wearable and wireless [26–29], so that doctors can examine patients from a distance. Auscultation became possible even while wearing personal protective equipment when treating patients with infectious diseases such as Coronavirus disease-19 (COVID-19) [30–32].

In this review, we will check the limitations of the existing auscultation method by checking the types of abnormal breathing sounds and the accuracy of analysis through the existing stethoscope. Next, we will introduce the new auscultation methods developed so far (AI-assisted analysis and wireless or attached stethoscopes) and the current status of breath sound analysis using them. Furthermore, we will suggest further research directions in the future.

### Classification of abnormal respiratory sounds

Respiratory sounds are produced by the airflow in the respiratory tract and are divided into two categories: Normal or abnormal sound. Normal respiratory sound is made when there is no pulmonary disorder and consist of tracheal, bronchial, bronchovesicular, and vesicular sounds [33]. Abnormal respiratory sounds are caused by diseases at the lung or bronchus [34]. They can be described by the mechanism of production, location they are detected in, characteristics (such as continuity, range of pitch, timing mostly heard), and acoustic features (Table 1) [35].

**Table 1:**
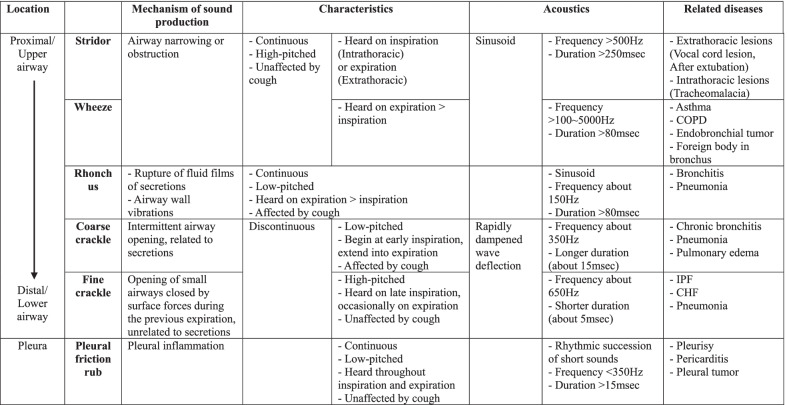
Classification of abnormal lung sounds and related diseases

Crackles are short, discontinuous, explosive sounds heard during inspiration and sometimes on expiration [36, 37]. Fine crackles are generated by inspiratory opening of small airways and associated with interstitial pneumonia or idiopathic pulmonary fibrosis (IPF), and congestive heart failure (CHF) [38]. Coarse crackles are produced by gas passing through intermittent airway opening and are related to secretory diseases such as chronic bronchitis and pneumonia [39].

Wheezes are generated in the narrowed or obstructed airway [36]. They have high frequency (> 100–5000 Hz) and sinusoidal oscillation in sound analysis [40]. They usually occur in obstructive airway diseases like asthma and chronic obstructive pulmonary disease (COPD) [39]. Rhonchi are induced by the narrowing of airways, caused by the production of secretions, so rhonchi can disappear after coughing (Table 1) [36].

Stridor is a high-pitched, continuous sound produced by turbulent airflow through a narrowed airway of upper respiratory tract [36]. It is usually a sign of airway obstruction that requires prompt intervention. In patients with pleural inflammation such as pleurisy or pleural tumor, a visceral pleura becomes rough, and its friction with the parietal pleura generates crackling sounds, a friction rub (Table 1) [41].

Although respiratory sounds are not difficult for a trained clinician to discern in usual cases, some sounds are ambiguous even for an expert to distinguish accurately. In addition, there are some cases where a mixture of several abnormal breathing sounds can be heard. Novel AI-assisted stethoscope can be useful for these challenging situations.

### Limitation of conventional stethoscope and auscultation

As mentioned earlier, inherent subjectivity is considered as the largest drawback of auscultation. Many studies have been performed to assess the human’s ability to auscultate and identify respiratory sounds (Table 2).

**Table 2:** Accuracy of human auscultation

Topic of study	Results	References
The accuracy of lung auscultation	<Correct detection rate by sounds>	[42]
	– Pulmonologists: 28% (abnormal bronchial sound), 90% (wheezes)	
	– Pediatricians: 16% (abnormal bronchial sound), 83% (wheezes)	
	– Interns: 13% (abnormal bronchial sound), 83% (wheezes)	
Physicians’ classification of lung sounds from video recordings	<Multirater agreement (Fleiss’ κ) between observers>	[43]
	– Detailed categories: 0.04 (rhonchi), 0.43 (high-pitched wheezes)	
	– Combined categories: 0.59 (wheezes), 0.62 (crackles)	
Pulmonary auscultatory skills during training in internal medicine and family practice	<Identification rates by sounds>	[44]
	– Trainees of family practice: 0% (whispered pectoriloquy), 84% (expiratory wheeze)	
	– Trainees of internal medicine: 1% (whispered pectoriloquy), 82% (expiratory wheeze)	
	– Pulmonary fellows: 5% (whispered pectoriloquy), 100% (expiratory wheeze)	
Comparing the auscultatory accuracy of health care professionals	<Correct detection rates by sounds>	[45]
	– Staff of internal medicine: 86.7% (wheezes), 96.7% (crackles)	
	– Resident of internal medicine: 59.0% (crackles), 80.0% (wheezes)	
	– Adult ICU nurses: 47.0% (crackles), 88.0% (wheezes)	
The contribution of spectrogram for visualization sound in clinical practice	<Proper diagnosis rate of medical students>	[46]
	Normal sounds: 57% (sound) → 63% (plus spectrogram)	
	Wheezes: 70% (sound) → 83% (plus spectrogram)	
	Crackles: 53% (sound) → 70% (plus spectrogram)	
	Stridor: 70% (sound) → 73% (plus spectrogram)	

Hafke-Dys et al. conducted a study comparing the skills of doctors and medical students in the auscultation of respiratory sounds. The pulmonologists performed remarkably better than the other groups and there was no significant difference in the rest of the groups [42]. Melbye et al. proceeded a study assessing the inter-observer variation in pediatricians and doctors for adults when classifying respiratory sounds into detailed or broader categories. The results indicated that descriptions of auscultation sounds in broader terms were more steadily shared between participants compared to more detailed descriptions [43]. Mangione et al. conducted a research assessing auscultatory skills of respiratory sounds among doctors and medical students. On average, trainees of internal medicine and family practice did not show significantly better performance than medical students. On the other hand, pulmonary fellows recorded the highest scores in all categories [44]. Mehmood et al. assessed the auscultatory accuracy of health care professionals working in medical intensive care unit (ICU). The sounds presented were wheezes, stridors, crackles, holosystolic murmur, and hyperdynamic bowel sounds. As expected, attending physicians performed best, followed by residents and subsequently nurses [45]. Andres measured the accuracy of medical students’ auscultation and investigated the efficacy of adding visual representation of sounds to support diagnosis and education. The results showed the potential of sound representation for increasing the accuracy of auscultation [46].

Overall, the studies have shown discrepancies in auscultation ability among doctors (especially in detailed classifications of respiratory sounds), suggesting that they may cause inaccurate diagnosis or incorrect treatment [47]. To reduce the subjective interpretation of sounds and complement the gap of auscultation capabilities between doctors, it would be helpful to establish a system which can record and share auscultated sounds.

Another drawback of auscultation is the impossibility of remote care. When doctors examine patients with a stethoscope, auscultation must be implemented by contacting the stethoscope on the body of patients. Many patients with chronic diseases or limited mobility stay in nursing facilities or at home often without a medical practitioner. Also, the demand of patients in hard-to-reach area for telemedicine is increasing nowadays. However, it is difficult for doctors to examine these patients and auscultation is hardly done. If a stethoscope that is easy to use even for non-specialists is developed using data transmission technology, doctors will be able to check the patient's condition from a distance.

### Deep-learning based analysis of respiratory sounds

Development of a standardized system to analyze respiratory sounds accurately is required to overcome the subjectivity of human auscultation and the discrepancy in auscultation ability between doctors [8]. Recently, machine learning-based AI techniques are applied mainly by deep learning networks in many areas including chest radiograph or electroencephalography (EEG) [48–50]. These AI techniques enable us to obtain a new approach or more accurate analysis of respiratory sounds [9]. In order to satisfy the requirement, there have been many attempts to develop a new method of classifying and interpreting respiratory sounds automatically using deep learning-based analysis [10, 11]. However, because of the black box type algorithmic property of the deep learning algorithm, there is a certain lack of interpretability of detailed information of the analysis [51]. Though interpretability is an important factor for analysis, it is highly related to technical issues and data dependency. Moreover, it is not clearly defined nor stable yet [51]. For this reason, interpretability will be not covered in this review.

From the machine learning perspective, there are two main parts for respiratory sound analysis. The first is to develop predictive algorithms or models based on well-known machine learning methods (support vector machine [SVM], K-nearest neighbors [KNN], artificial neural network [ANN]) and deep learning architectures (convolutional neural networks [CNN], residual networks [ResNet], long short-term memory [LSTM], gated recurrent unit [GRU]) with multi-layers and the second is to define appropriate features explaining respiratory sound characteristics and extract them (short-time Fourier-transformed [STFT], wavelet transform [WT], Mel-frequency cepstrum coefficient [MFCC], singular spectrum analysis [SSA]) from given data and their ensembles. In this point of view, methods and algorithms for respiratory sound classification and prediction are summarized in more detail below (Table 3).

**Table 3:** Deep learning-based analysis of respiratory sounds

Topic of study	Number of subject/recording	Number of classes	Applied model	Result		References
Recognition of pulmonary diseases from lung sounds using CNN and LSTM	213/1483	6: Normal, asthma, pneumonia, bronchiectasis, COPD, heart failure	CNN, biLSTM	Accuracy		[12]
				biLSTM: 98.16%		
				CNN: 99.04%		
				CNN + biLSTM: 99.62%		
Classification of respiratory sounds using OST and deep residual networks	Not available/489	3: Crackle, wheeze, normal	OST, ResNets	Accuracy		[13]
				STFT: 93.98%		
				ST: 97.79%		
				OST + ResNets: 98.79%		
Detection of respiratory sounds based on wavelet coefficients and machine learning	130/705	3: Crackles, rhonchi, normal	SVM, ANN, KNN	Accuracy		[14]
				SVM: 69.50%		
				ANN: 85.43%		
				KNN: 68.51%		
Benchmarking of eight recurrent neural network variants for breath phase and adventitious sound detection	279/9765	6: Inhalation, exhalation, wheeze, stridor, rhonchus, crackles	LSTM, GRU, BiLSTM, BiGRU^c^, CNN-LSTM, CNN-GRU, CNN-BiLSTM, and CNN-BiGRU	F1 score		[15]
				LSTM: 73.9%	GRU: 77.6%	
				BiLSTM: 76.2%	BiGRU: 78.4%	
				CNN-LSTM: 78.1%	CNN-GRU: 80.6%	
				CNN-BiLSTM: 80.3%	CNN-BiGRU: 80.6%	
Classification of lung sounds through DS-CNN models with fused STFT and MFCC features	Not available/12,691	4: Normal, wheeze, crackle, unknown	DS-CNN, VGG-16, AlexNet, DS-AlexNet, LSTM, GRU, TCN^d^	Accuracy		[16]
				DS-CNN: 85.74%	VGG-16: 85.66%	
				AlexNet: 79.92%	DS-AlexNet: 80.86%	
				LSTM: 76.92%	GRU: 78.50%	
				TCN: 75.51%		
Implementation of AI algorithms in pulmonary auscultation examination	50/522	4: Wheezes, rhonchi, fine and coarse crackles	Neural network	F1-score (%)		[17]
				Coarse crackles: Doctors (42.8%), NN (47.1%)		
				Fine crackles: Doctors (51.1%), NN (64.6%)		
				Wheezes: Doctors (61.8%), NN (66.4%)		
				Rhonchi: Doctors (61.0%), NN (72%)		
AI accuracy in detecting pathological breath sounds in children	25/192	2: Crackles, wheeze	Neural network	PPA^a^/NPA^b^		[18]
				Crackles: Clinicloud 0.95/0.99, Littman 0.82/0.96		
				Wheezes: Clinicloud 0.90/0.97, Littman 0.80/0.95		
Classification of lung sounds using CNN	1630/17,930	① 2: Healthy, pathological	CNN, SVM	Accuracy (CNN/SVM)		[19]
		② 3: Rale, rhonchus, normal		① 86%/86%	② 76%/75%	
Feature extraction technique for pulmonary sound analysis based on EMD	30/120	3: Normal, wheezes, crackles	ANN, SVM, GMM	Accuracy		[20]
				EMD with ANN: 94.16%		
				EMD with SVM: 93.75%		
				EMD with GMM: 88.16%		
Application of deep Learning to classify the severity of COPD	Not available/120	2: Crackles, wheeze	NN, DBN	Accuracy		[21]
				NN: 77.60%		
				DBN: 95.84%		
Application of deep Learning to detect early COPD	50/600	1: Wheeze	DBN	Accuracy		[22]
				DBN: 93.67%		
Application of semi-supervised deep learning to lung sound analysis	284/11627	2: Crackles, wheeze	SVM	AUC		[23]
				Crackle: 0.74		
				Wheeze: 0.86		

^a^*PPA* positive percent agreement

^b^*NPA* negative percent agreement

^c^*BiGRU* bidirectional gated recurrent unit

^d^*TCN* temporal convolutional network, *DBN* deep belief networks, *COPD* chronic obstructive pulmonary disease

Fraiwan et al. conducted a study to explore the ability of deep learning algorithms in recognizing pulmonary diseases from recorded lung sounds. After several preprocessing steps (wavelet smoothing, displacement artifact removal, and z-sore normalization), two deep learning network architectures including CNN and bidirectional long short-term memory (biLSTM) units were applied. The resulting algorithm (CNN + biLSTM) achieved the highest accuracy [12]. Chen et al. proceeded with research to overcome the limitations of existing classification methods of lung sounds; artifacts and constrained feature extraction methods. The proposed method using optimized S-transform (OST) and deep ResNets outperformed the ensemble of CNN and the empirical mode decomposition (EMD)-based ANN [13]. Meng et al. combined the wavelet signal similarity with the relative wavelet energy and entropy as the feature vector to extract features of lung sounds. Applying the ANN to this system showed higher accuracy than the methods using SVM and KNN [14]. Hsu et al. applied eight kinds of AI-technique models and conducted a performance comparison between them. GRU-based models outperformed the LSTM-based models, and bidirectional models outperformed unidirectional counterparts. Moreover, adding CNN improved the accuracy of lung sounds analysis [15]. Jung et al. proposed a feature extracting process through the depthwise separable-convolution neural network (DS-CNN) to classify lung sounds accurately. Also, they found that the fusion of the STFT and the MFCC features and DS-CNN achieved a higher accuracy than other methods [16]. Grzywalski et al. compared the efficiency of auscultation of doctors and machine learning-based analysis based on neural networks and proposed that the efficiency could be improved by the implementation of automatic analysis [17]. Kevat et al. showed that a neural network-based AI algorithm detected respiratory sounds with a high accuracy [18]. Aykanat et al. found that CNN and SVM machine learning algorithms can be used to classify lung sounds, but the accuracy decreased as the number of sounds to be compared increased, as with humans [19]. Mondal et al. proposed a feature extraction technique based on EMD improving the performance of lung sound classification and the method was compared with WT, MFCC, and SSA method-based classification systems including ANN, SVM, and Gaussian mixture model (GMM) classifier. The proposed method gives a higher accuracy of 94.16 for an ANN classifier [20]. Altan applied deep belief networks (DBN) algorithm to diagnose early COPD and classify the severity of COPD, and the results showed significantly high accuracy. Since COPD is irreversible when it progresses, early diagnosis is important. In this regard, the results of their studies are groundbreaking and useful [21, 22]. Chamberlain et al. applied SVM with a semi-supervised deep learning algorithm and their algorithm achieved receiver operating characteristic (ROC) curves with a relatively high area under the ROC curve (AUC) [52].

Many studies have been conducted in collaboration with doctors and machine learning experts, and it has become possible to discriminate lung sounds with a considerable level of accuracy. However, there is still a limitation that the analysis becomes less accurate when noises caused by the stethoscope itself, surrounding environment, other organ activities, and so on are mixed among the recorded sounds or when two or more breathing sounds are present at the same time. This should be resolved through additional research in the future [53].

### Development of digital stethoscopes

There are several available electronic stethoscopes: Littmann 3100, Stethee pro, Thinklabs one digital amplified medical stethoscope, Littman core digital stethoscope 8490, and StethoMe (Table 4). These digital stethoscopes overcome the low sound levels by electronically intensifying the respiratory sounds. Most importantly, recording of respiratory sounds with a digital stethoscope has allowed and facilitated the study of automatic respiratory sound analysis. Littmann 3100 is one of the most popular electronic stethoscopes, and many studies using respiratory sounds have been conducted with this stethoscope [54, 55]. It can save multiple sounds and transmit the data via Bluetooth transmission. Interestingly, Stethee Pro uses machine learning algorithms to capture and monitor both heart and lung sounds. This stethoscope can amplify the sound up to 96 times and visualize the sound data on the screen. Thinklabs One is the smallest digital stethoscope, and it can be used for personal protective equipment (PPE) auscultation in patients with infectious diseases such as COVID-19. StethoMe was developed for homecare service and installed AI can analyze the abnormality of respiratory sound. It is particularly specialized for monitoring airway diseases including asthma. These digital stethoscopes are continuously developing and becoming more useful for monitoring and diagnosing pulmonary disease.

**Table 4:** Developing stethoscopes: digital, wireless, or wearable device

Model/study	Characteristics	Manufacturer/references
Littmann 3100 Electronic Stethoscope	24× amplification	Littmann^®^
	Record and save	
	Bluetooth transmission	
Stethee Pro	96× amplification	M3DICINE Inc^®^
	Machine learning algorithm	
	Ambient noise cancellation	
Thinklabs One Digital Amplified Medical Stethoscope	100× amplification	Thinklabs One^®^
	Precision filtering	
	Personal protective equipment auscultation	
StethoMe	Homecare service	StethoMe^®^
	AI analyses the respiratory sounds	
A wearable stethoscope for long-term ambulatory respiratory health monitoring	Long-term ambulatory	[26]
	Respiratory health monitoring	
	Diaphragm-less acousto-electric transducer	
Wearable multimodal stethoscope patch	Wearable biosignal acquisition	[27]
	High quality cardiac and pulmonary auscultation	
Wearable cardiorespiratory monitoring	Estimation of respiration using a phonocardiogram	[28]
Epidermal mechano-acoustic electrophysiological measurement device	Water-permeable, adhesive, biocompatible, and reversible device	[29]

In addition, recent innovative advances in battery technology, embedded processors with low power consumption, and integrated sensors have made many medical devices wearable and wireless (Table 4). Some studies have applied these techniques to stethoscopes, and the researchers developed the stethoscopes that monitor cardiorespiratory signals through wireless bio-signal acquisition [26, 27]. Certain airway diseases, such as asthma, often get worse at night or early in the morning, so doctors often cannot detect them during the daytime. Just as in the diagnosis of arrhythmia disease, Holter monitoring is used to monitor a patient's heart rate for 24 h, continuous monitoring of respiratory sound through a wearable device in airway disease will be of great help in diagnosis and emergency treatment. Some groups developed water permeable, adhesive, biocompatible acoustic devices for electrophysiological recording [28, 29]. Technologies of recording of sounds clearly and filtering out noises need further improvement, but wearable stethoscopes are expected to be used to diagnose and monitor chronic pulmonary diseases soon.

### Clinical application of digital stethoscopes and AI-assisted analysis

There are several clinical studies using distal stethoscopes and AI for respiratory analysis. One study showed that CNN can classify chronic disease, non-chronic disease, and healthy groups by automatically analyzing respiratory sounds. In addition, the CNN is able to subcategorize disease group to different types of diseases including COPD, bronchiectasis, pneumonia, and bronchiolitis (Table 5) [56]. Another study adopted the acoustic characteristics of fine crackles to predict honeycombing on chest computed tomography (CT). They concluded that the presence of honeycombing was independently associated with onset time, number of crackles in the inspiratory phase, and F99 value of fine crackles [57].

**Table 5:** Clinical trials of novel digital stethoscope and AI-assisted analysis

Topic of study	Results/characteristics	Condition/disease Study type (status)	Reference and ClinicalTrial.gov. identifier
Detecting respiratory pathologies using CNN and variational autoencoders	CNN was used to classify chronic disease, non-chronic disease and healthy group	Chronic disease	[56]
		Non-chronic disease	
		Healthy	
		Observational (completed)	
Predicting honeycombing on HRCT by the acoustic characteristics of fine crackles	Acoustic properties of fine crackles distinguish the honeycombing from non-honeycombing group	Honeycombing	[57]
		Non-honeycombing	
		Observational (completed)	
Diagnosing interstitial pneumonia by analyzing inspiratory lung sounds recorded with phonopneumography	Spectral analysis of lung sounds is useful in the diagnosis and evaluation of the severity of IP	Interstitial pneumonia	[59]
		Healthy	
		Observational (completed)	
Evaluating Auscul-X, a Touch Free Digital Stethoscope	Multichannel, touch-free electronic stethoscope	COVID-19	NCT04570189
		Observational (recruiting)	
Collecting respiratory sound samples to diagnose COVID-19 patients	VOQX Electronic Stethoscope	COVID-19	NCT04910191
	Sound signals are processed by machine learning algorithm	Interventional (recruiting)	
Clinical Evaluation of Automatic Classification of Respiratory System Sounds	StethoMe stethoscope	Wheezing, rhonchi, crackle, lung sound	NCT04208360
	AI software application	Observational (not yet recruiting)	
Digital auscultation test—IPF data collection	Littmann Digital Stethoscope	Idiopathic pulmonary fibrosis	NCT03503188
	3M Littmann Steth Assist software	Interventional (completed)	
Respiratory auscultation of an open real-time tele-stethoscope system	Open real-time tele-stethoscope system	Respiratory/heart disease	NCT03596541
	Respiratory crackle	Interventional (not yet recruiting)	

Many studies related to digital stethoscope and AI analysis of auscultation sound are still currently in progress. As the need to collect and analyze the auscultation sounds of patients in quarantine facilities increases due to the recent COVID-19 crisis, related research is being conducted more actively. Several studies are trying to find a typical pattern of auditory sounds in COVID-19 patients (Table 5). One study plans to evaluate the AI-aided auscultation with automatic classification of respiratory sounds by using StethoMe stethoscope. If these studies are conducted well and AI-equipped stethoscopes can detect wheezing, rhonchi, and crackle accurately, these stethoscopes will be useful in emergency room treatment, medical screening, and telemedicine fields [58]. These smart stethoscopes will be of great help in monitoring patients with chronic pulmonary diseases, and many studies are underway for patients with idiopathic pulmonary fibrosis (IPF) and COPD (Table 5).

## Conclusion

Thanks to the development of digital stethoscope and sound transmission technology, we have already been able to record and share respiratory sounds. With deep learning-based breathing sound analysis algorithm, we can distinguish respiratory sounds to some extent without a pulmonologist. This makes it possible to overcome the subjectivity in interpretation of sounds, the biggest drawback of the stethoscope, and this smart stethoscope will help the rapid diagnosis and the choice of appropriate treatment methods of respiratory diseases.

**Fig. 1:**
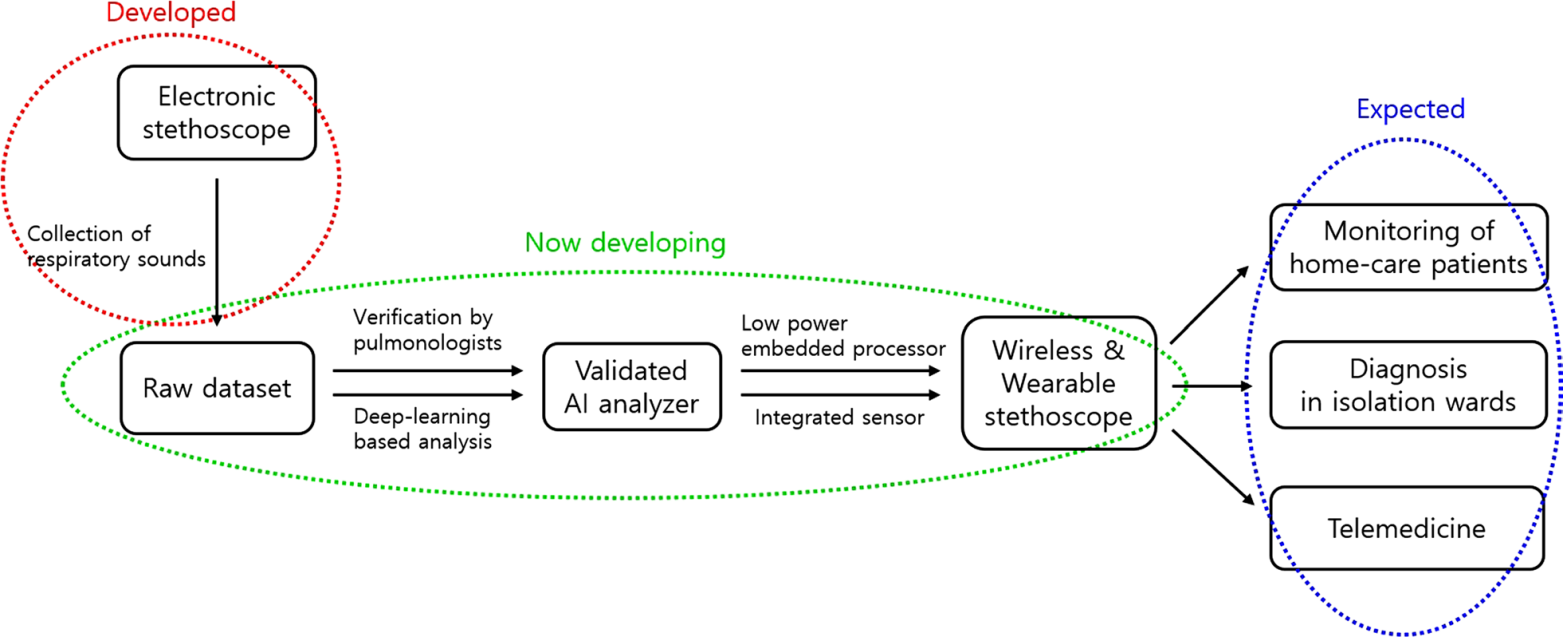
Summary of new medical era using smart stethoscope

In addition, current research on battery technology, embedded processors with low power consumption, and integrated sensors are expected to make stethoscopes and other medical devices wearable in addition to wireless. Through these advances, we will be able to get over another major limitation of the existing stethoscope, the impossibility of remote care. The latest medical demands such as non-face-to-face care due to COVID-19, the monitoring of chronic respiratory diseases, and telemedicine in the hard-to-reach area will be satisfied (Fig. 1).

However, despite the innovative developments so far, there are still some problems for the smart stethoscope to overcome. Since noises exist in the actual medical field where auscultation is performed, careful attention is required in recording and interpreting respiratory sounds. Noise filtering is one of the most crucial and challenging points in the aspect of both mechanical devices and analyzing algorithms. Although respiratory sounds are sometimes heard alone, in many cases, two or more sounds are mixed. These problems suggest the need for processing sound data acquired under noisy conditions to improve the sound quality. This would help rationally classify a wider variety of automatically auscultated sounds. Now, with the development of chest imaging, the degree of dependence on auscultation is relatively decreasing. However, as the remaining challenges are solved through further researches and clinical feedbacks, the smart stethoscope will become a definitely useful and essential tool in the diagnosis and treatment of respiratory diseases.

## Data Availability

Not applicable.

## Abbreviations

AI: Artificial intelligence
ML: Machine learning
COVID-19: Coronavirus disease-19
IPF: Idiopathic pulmonary fibrosis
CHF: Congestive heart failure
COPD: Chronic obstructive pulmonary disease
ICU: Intensive care unit
EEG: Electroencephalography
SVM: Support vector machine
KNN: K-nearest neighbors
ANN: Artificial neural network
CNN: Convolutional neural networks
ResNet: Residual networks
LSTM: Long short-term memory
GRU: Gated recurrent unit
STFT: Short-time Fourier-transformed
WT: Wavelet transform
MFCC: Mel-frequency cepstrum coefficient
SSA: Singular spectrum analysis
biLSTM: Bidirectional long short-term memory
OST: Optimized S-transform
EMD: Empirical mode decomposition
DS-CNN: Depthwise separable-convolution neural network
GMM: Gaussian mixture model
DBN: Deep belief networks
ROC: Receiver operating characteristic
AUC: Area under the ROC curve
PPE: Personal protective equipment
CT: Computed tomography
